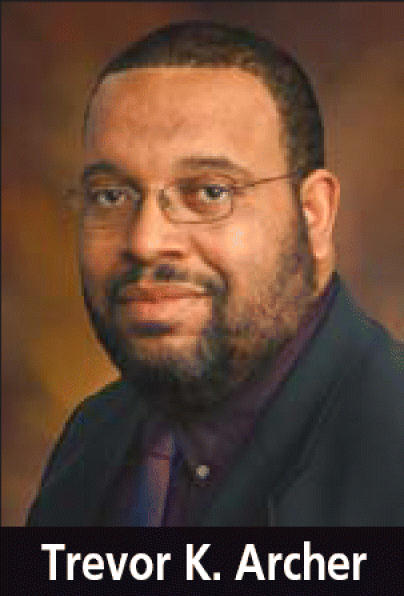# Guest Editorial: Epigenetics: Environmental Instructions for the Genome

**DOI:** 10.1289/ehp.114-a140

**Published:** 2006-03

**Authors:** Paul A. Wade, Trevor K. Archer

**Affiliations:** Laboratory of Molecular Carcinogenesis, National Institute of Environmental Health Sciences, National Institutes of Health, Department of Health and Human Services, Research Triangle Park, North Carolina, E-mail: archer1@niehs.nih.gov

Understanding how the environments we are exposed to influence the diseases we contract and the severity of those diseases is a major goal of the National Institute of Environmental Health Sciences (NIEHS). Although much effort has focused on the gene–environment axis, there is a growing body of information that suggests that environmental influences may extend beyond the DNA sequences of our genes. The recent completion of sequencing projects for the genomes of humans and several model organisms has provided, for the first time, a glimpse of the information required for the diversity of eukaryotic life. The information embedded in the linear nucleotide sequence of DNA contains coding information for RNA and protein, as well as regulatory sequences that control the biology of DNA itself. However, eukaryotic cells contain an additional level of information superimposed on the DNA double helix in the form of a complex nucleoprotein entity generically termed “chromatin.” Recent studies have highlighted the instructive nature of this “DNA packaging” in regulating the interactions of the enzymatic machines of replication, transcription, recombination, and repair with DNA. The emerging field dedicated to the study of this form of biologic regulation is termed “epigenetics.” Formally, epigenetics constitutes the study of changes in gene expression not accompanied by alterations in DNA sequence. In a more practical sense, epigenetics includes the study of the protein constituents of chromatin, the interaction of microRNAs with the genome, and the protein and DNA modifications that appear to define biologic states in local regions of chromosomes.

The field of epigenetics has existed for decades, springing from genetic studies of the phenotypic variability of diverse biologic readouts such as eye color in *Drosophila* (Muller 1930; Schultz 1950), mating cassette silencing in yeast (Hawthorn 1963), and X chromosome inactivation in mammals (Lyon 1961). These early genetic studies resulted in the establishment of functional relationships among various factors in regulation of gene expression through analysis of their genetic interactions. Biochemical identification and characterization of enzymatic machinery responsible for DNA modification (Bestor et al. 1988; Okano et al. 1998) and histone modification patterns (Brownell et al. 1996; Taunton et al. 1996) in the 1980s and 1990s led to a greater appreciation of the underlying biochemical principles of epigenetic regulation (Fischle et al. 2003; Rea et al. 2000). These studies relied heavily on prior genetic data and provided mechanistic insight into how genetic modifiers of eye color variegation, for example, regulate gene expression (Fischle et al. 2003; Rea et al. 2000).

A unifying concept central to a modern, molecular view of epigenetics postulates that the pattern of modifications (both of DNA and of histone proteins themselves) provides information content that instructs the enzymes integral to nuclear physiology (Jaenisch and Bird 2003; Strahl and Allis 2000). Importantly, although chromosomes are dynamic organelles, the epigenetic information can be quite stable, surviving multiple rounds of mitotic cell division (Cavalli and Paro 1998), meiosis (Cavalli and Paro 1998), or even nuclear transfer (Ng and Gurdon 2005). In a sense, the chromosome contains two intertwined types of information: the linear sequence of nucleotide bases in DNA codes for biologic macromolecules, and the regulatory information embedded in the nucleoprotein architecture of chromatin specifies which regions of the genome are active in any given cell. Epigenetic regulation is a primary driving force behind the creation of different cell types, each with the DNA sequence, during development of multicellular organisms. A seminal finding that monozygotic twins are epigenetically indistinguishable early in life but, with age, exhibit substantial differences in epigenetic markers underscores the important role played by the environment in shaping the epigenome (Fraga et al. 2005).

Given the importance of epigenetic regulation to normal nuclear function, it is pertinent to ask whether alterations in this form of regulation might impact disease. Components of the epigenetic machinery, in fact, are altered in various human diseases including neurologic disorders (e.g., Rett syndrome and α-thalassemia X-linked mental retardation), congenital malformation (e.g., Rubinstein Taybi syndrome), immune disorders (e.g., ICF syndrome), and even aging. Epigenetic alterations also constitute the molecular basis of pathology associated with loss of imprinting (e.g., Prader-Willi, Angelman, and Beckwith-Weidemann syndromes). Moreover, there are multiple connections between epigenetic errors and neoplasia including alterations in genomic DNA methylation (Ballestar and Esteller 2005) and histone acetylation patterns (Ballestar and Esteller 2005; Slany 2005).

The current excitement over epigenetics and its potential as a tool for diagnosis and treatment of human disease seems warranted. However, there are still important knowledge gaps in the field. The solution of the structure of DNA by Watson and Crick in the 1950s (Watson and Crick 1953) immediately suggested a mechanism—semiconservative replication—by which the genetic material could be faithfully transmitted from one generation to the next. A major dilemma for the field of epigenetics concerns how the regulatory information embedded in the protein constituents of chromatin is replicated during S phase. Identification of the mechanisms involved in faithfully copying the epigenetic information will represent an important conceptual advance. Additionally, we do not currently understand the “language” of epigenetics. Deciphering the genetic code revealed the language used by DNA to propagate information. The current state-of-the-art translation of epigenetic cues is both crude and limited in scope. Despite the considerable progress made in this area over the past decade, scientists are only beginning to decipher the information embedded in chromatin. The alphabet of epigenetics is not completely described and, importantly, the mechanism by which cells read and interpret this information is also largely unknown.

Epigenetic information promises to serve as an important adjunct to DNA sequence in the analysis of biologic response to environmental exposures. Obviously, biologic parameters that contribute to the functionality of DNA will be affected by exposure in much the same manner as DNA sequence (by mutation). A major difference between epigenetic and genetic outcomes is that while DNA sequence is static, the epigenome is a dynamic entity that changes with cell type, during the cell cycle, in response to biologic signaling systems, and with environmental changes. Deciphering how the epigenome responds to environmental exposures and how it predicts disease risk holds great promise and will undoubtedly prove an important adjunct to mutational analyses. Recently, multiple reports have linked epigenetic mechanisms to the phenotypic effects of environmental exposures during critical periods of development (Anway et al. 2005). In real human terms, these exposures result from such variable behaviors as nutrition and lifestyle. Some of these may have a direct influence on embryonic development, whereas others may exert their effects later in life, as predicted by the Barker hypothesis (Barker 1990).

Although current understanding of epigenetics lags behind our more fundamental understanding of the information content in DNA sequence, the epigenome will undoubtedly serve as a therapeutic target in the future. In fact, epigenetic therapies are currently under investigation in diverse diseases including cancer, sickle cell anemia, and thalassemias (de Vos 2005). Personalized medicine will certainly be affected by epigenetic differences between individuals and the contributions of this epigenetic polymorphism to disease onset, severity, and outcome. The seemingly limitless potential applications to problems relevant to human health and disease underscore the need to elucidate the basic principles of epigenetic regulation at a molecular level. Understanding the mechanistic basis of how epigenetic regulation is achieved is fundamental to placing this level of biologic regulation in the tool box of environmental health science and medicine.

## Figures and Tables

**Figure:**
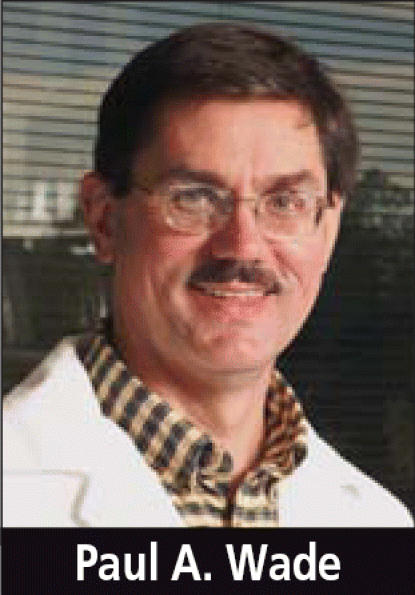


**Figure:**